# Transformer-Based Vehicle-Trajectory Prediction at Urban Low-Speed T-Intersection

**DOI:** 10.3390/s25144256

**Published:** 2025-07-08

**Authors:** Jae Kwan Lee

**Affiliations:** Department of Highway & Transportation Research, Korea Institute of Civil Engineering and Building Technology, 283 Goyangdae-ro, Goyang-si 10223, Republic of Korea; jklee645@kict.re.kr

**Keywords:** vehicle-trajectory, T-intersection, transformer model, urban driving, trajectory forecasting, urban driving scenarios, microscopic traffic simulation

## Abstract

Transformer-based models have demonstrated outstanding performance in trajectory prediction; however, their complex architecture demands substantial computing power, and their performance degrades significantly in long-term prediction. A transformer model was developed to predict vehicle trajectory in urban low-speed T-intersections. Microscopic traffic simulation data were generated to train the trajectory-prediction model; furthermore, validation data focusing on atypical scenarios were also produced. The appropriate loss function to improve prediction accuracy was explored, and the optimal input/output sequence length for efficient data management was examined. Various driving-characteristics data were employed to evaluate the model’s generalization performance. Consequently, the smooth L1 loss function showed outstanding performance. The optimal length for the input and output sequences was found to be 1 and 3 s, respectively, for trajectory prediction. Additionally, improving the model structure—rather than diversifying the training data—is necessary to enhance generalization performance in atypical driving situations. Finally, this study confirmed that the additional features such as vehicle position and speed variation extracted from the original trajectory data decreased the model accuracy by about 21%. These findings contribute to the development of applicable lightweight models in edge computing infrastructure to be installed at intersections, as well as the development of a trajectory prediction and accident analysis system for various scenarios.

## 1. Introduction

In line with the recent advances in autonomous driving technology, urban transportation systems require accurate trajectory-prediction technology for vehicle interactions and accident prevention in complex and diverse driving environments. Particularly, urban low-speed T-intersections frequently face complex driving trajectories with straight and turning driving patterns, as well as unpredictable and atypical situations such as vehicle deceleration and abrupt stops. Prior studies have demonstrated that predicting vehicle trajectories can significantly help in preventing collisions on the road. In particular, edge computing-based approaches for accident detection and collision avoidance underscore the need for highly accurate trajectory forecasts [1,2]. Additionally, vehicles approaching an intersection must overcome visibility obstructions caused by roadside buildings or parked vehicles and must comply with yield or priority arrival rules when entering the intersection, posing special challenges to trajectory prediction. Therefore, technology that accurately predicts the future position of vehicles in such environments is essential for collision prevention and efficient traffic flow control and plays an important role in creating a smart transportation infrastructure.

Previous trajectory-prediction studies have mainly utilized recurrent neural network-based models [3,4,5,6,7,8,9]; however, recently, transformer-based models [10] have been highlighted due to their capability to efficiently handle long sequences [11,12,13,14,15,16,17,18]. Transformer models can more effectively capture long-term temporal dependencies through self-attention mechanisms; moreover, they are able to handle inputs and outputs of various lengths [10]. However, their prediction performance may degrade drastically as their long-term prediction length increases [12,16,18]. Thus, an efficient model design that can minimize performance degradation is required for practical utilization in real road environments.

A transformer-based vehicle trajectory-prediction model was designed and optimized to address this challenge and improve trajectory-prediction accuracy at urban low-speed T-intersections. While the ultimate goal is to develop trajectory-prediction models based on real-world driving data, this study focuses on the preliminary stage of model development by using synthetic data generated from a microscopic traffic simulation. The use of simulation enables controlled experiments and iterative testing of model design strategies, which can serve as a foundation for future domain-adaptive modeling using real field data. Four experiments were conducted. First, the models were trained with mean squared error (MSE), smooth L1, and combined loss, and their performance was compared and analyzed to determine the optimal loss function. Second, optimal model configurations were identified by exploring the most effective input and output sequence lengths for prediction performance. Third, for each driving pattern, such as speed deceleration/acceleration, stop-and-go, and uniform motion, simulation data were generated, and the prediction performance in these special situations was evaluated to assess generalization capability. Finally, the performance was evaluated when extending the input feature to include additional variation features.

The following results were obtained: (1) the transformer-based model performed best for turning prediction when a smooth L1 loss function was applied; (2) by using trajectory length of about 1 s is advantageous in reducing prediction errors; (3) additional training on special situations, such as stop-and-go, showed the possibility of improving the generalization performance, which can cope with various driving characteristics; however, simple feature extension degraded the model performance. Therefore, the model structure and design strategy proposed in this study are expected to contribute to the advancement of vehicle-trajectory-prediction technology at urban T-intersections and to be practically applied to future autonomous driving and transportation infrastructure systems.

This paper is organized as follows. Section 2 reviews related studies. Section 3 describes the methodology for generating trajectory data and the trajectory prediction model design. Section 4 presents the evaluation results. Section 5 presents the results of the performance evaluation of the model on scenario-specific trajectory data. Finally, conclusions are presented in Section 6.

## 2. Related Works

Prediction of vehicle trajectories at urban low-speed T-intersections is important for ensuring road safety by assisting both autonomous vehicles and human drivers. However, there is a difference regarding the perspective of the trajectory. A strand of research predicts the trajectories of surrounding objects to recognize the surrounding situations from the vehicle perspective, aiming to generate safe driving paths for vehicles [19,20,21]. Another research strand predicts trajectories from the road perspective, aiming to identify the movement of all objects on the road and ensure that all objects move safely [11,14,15]. Studies that identify object movement and predict future trajectories from the road perspective were reviewed.

Previous trajectory-prediction studies [3,4,5,6,7,8,9] employing deep learning have chiefly utilized recurrent neural networks (RNNs) [22], long short-term memory (LSTM) networks [23], and gated recurrent units (GRUs) [24]. After RNN-based trajectory-prediction methods were developed using existing trajectory data, methodologies have been proposed to automate feature extraction [7,8,25] and predict trajectories over long periods [25], as an end-to-end model combined with convolutional neural networks [26]. A model combining a seq2seq mechanism [27] and LSTM networks was developed, showing an accuracy improvement of approximately 20%, compared with simple LSTM, GRU, and other models [28]. However, it had the disadvantage of requiring longer training time by more than 30%, compared with simple LSTM [28]. RNNs can learn a vehicle’s dynamic changes over time [3,4,5] but have limitations in information loss or learning inefficiency when processing long-sequence data.

In recent studies, transformers have been widely utilized due to their outstanding performance in processing time series data [11,12,13,14,15,16,17,18]. Similarly, studies on pedestrian trajectory prediction have shown that transformer-based models have outperformed RNN models [29,30]. Transformer models show high performance because they can learn interactions between all locations within a sequence in parallel by utilizing self-attention and can effectively capture long-term dependencies [10]. As for recent studies that have utilized transformer models for vehicle-trajectory prediction, Singh and Srivastava [13] presented a method for extracting multi-scale spatial features from trajectory data graphs and a transformer model trained on them, achieving a final displacement error (FDE) of 0.69 m on the Argoverse dataset [31]. Meanwhile, Amin [11] developed a method of extracting vehicle trajectories from the NGSIM dataset [32], as well as a transformer-based model; by comparing the model’s performance with those of LSTM and GRU-based models, the trajectory-prediction accuracy improved by a maximum of 57%. Furthermore, Zhang [14] developed a model that sequentially predicted trajectories after extracting trajectory data from four-legged intersection data from the Lyft dataset [33]; in the trajectory-prediction process, the transformer-based model showed an accuracy improvement higher than 40%, compared with LSTM and GRU-based models. As such, transformer-based trajectory-prediction models can be expected to outperform RNN-based models. Furthermore, the scalability of considering interactions between vehicles or predicting multiple vehicle trajectories simultaneously has been proposed [11,15].

Advances in trajectory-prediction research are expected to improve performance; however, problems regarding long-term prediction and generalization performance have been identified as issues. In studies on transformer-based trajectory-prediction models, the prediction error tended to increase as the trajectory length predicted increased [11,12,16,18]. When evaluating transformer models on the NGSIM dataset [32], it has been found that their error increased a maximum of 12 times when predicting long-term trajectories, compared with predicting short-term trajectories [11,12]. Such performance degradation is relevant to the characteristics of the transformer structure, such as distraction of attention and dilution of information at the input point [16]. Thus, it is necessary to identify the length at which reliable predictions can be made in order to use the developed model in the field. Furthermore, an appropriate input sequence length for trajectory prediction also determines the prediction performance of the model [34,35]. Another important problem for trajectory-prediction models is their generalization performance—how well they cope with road geometry and vehicle driving patterns not included in the training [36]. Beyond simply improving the average accuracy, it is also important to evaluate the prediction performance in rare situations to increase its applicability in real situations. Previous studies have assessed the generalization capability of models using test data, including various scenarios, which has been recognized as an important indicator of model applicability in real-world road environments [11,15].

Table 1 shows the strengths and limitations of three major deep learning architectures for trajectory prediction. In this study, the transformer architecture was selected for trajectory prediction due to its ability to capture long-term dependencies and enable parallel sequence processing. This offers advantages over traditional recurrent networks (RNN/LSTM/GRU), which often struggle with very long sequences and can suffer from vanishing gradient issues. Thus, the transformer architecture is well-suited to learn complex driving patterns over extended time horizons. The outstanding characteristics of transformer architecture were leveraged while its performance was improved by reflecting the complex driving characteristics of urban low-speed T-intersections. Particularly, it attempted to develop a lightweight model that can be practically applied in road infrastructure, as well as present a more practical model design through generalization performance evaluation for various scenarios.

## 3. Methodology

**Figure 1:**
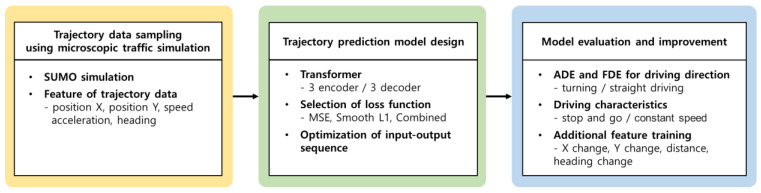
Experimental design of this study.

Figure 1 summarizes the methodology of this study. First, trajectory data were generated using the microscopic traffic simulation. Second, the transformer models were trained on the generated trajectory data. To determine a model structure that improves the prediction performance and the learning methodology, the model structures were designed via two experiments: Experiment (1) compared the performance of different types of loss functions, while Experiment (2) compared the performance of the model according to the length of the input–output sequence. Third, the performance of the trained models was evaluated according to the driving scenario, aiming to explore the direction of performance improvement. Finally, the performance improvement of the model was evaluated by utilizing new feature data extracted from the trajectory data.

### 3.1. Trajectory Data Sampling

**Figure 2:**
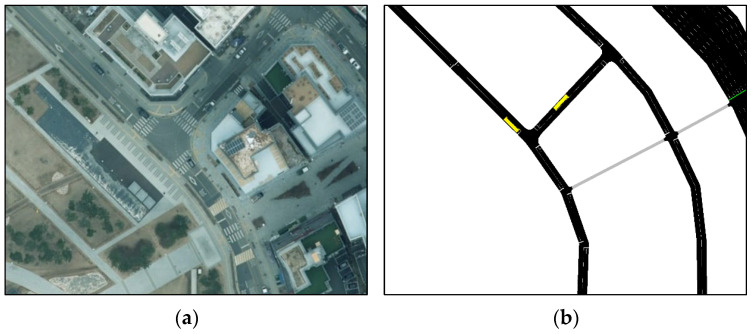
The simulation site for trajectory data sampling: (**a**) the simulation site and (**b**) the SUMO simulation network.

To generate vehicle-trajectory simulation data for training the model, a real city road was selected. A microscopic traffic simulation program, SUMO [37], was utilized for the simulation. Since this study aimed to develop a trajectory-prediction model for urban T-intersections, a three-legged unsignalized T-intersection with two lanes was selected. The speed limit of the intersection was set to 30 km/h because the selected T-intersections are densely populated with parks and general commercial facilities, and there is a high risk of accidents due to the high number of pedestrians and vehicles. However, the trajectory data for pedestrians were not generated; only vehicle behavior was simulated to generate trajectory data for vehicles. Figure 2 presents the locations where the simulation was implemented and the networks created by SUMO. The Krauss model [38] was utilized as a car-following model to determine vehicles’ behavior, and the randomTrip function provided by SUMO was employed to allow vehicles to travel over the entire network.

A car-following model is a methodology that models the behavior of the driver of a car following the longitudinal movement of a vehicle in the presence of more than two vehicles. Representative car-following models include the Wiedemann [39], Krauss [38], and intelligent driver [40] models. In this study, the Krauss model was used. The Krauss model [38] sets the target velocity ($v_{des}$) of the vehicle based on the safe velocity ($v_{safe}$) and determines the behavior of the vehicle at the current time. The safe velocity can be calculated by Equations (1) and (2).

(1) $$\begin{array}{c} v_{safe}=v_{n-1}\left(t\right)+\frac{g_{n}\left(t\right)-v_{n}\left(t\right)\tau_{k}}{\frac{v_{n-1}\left(t\right)+v_{n}\left(t\right)}{2b}+\tau_{k}} \end{array}$$

(2) $$\begin{array}{c} g_{n}\left(t\right)=x_{n-1}\left(t\right)-x_{n}\left(t\right)-s\; \end{array}$$

where $v_{n-1}\left(t\right)$ is the velocity of the vehicle in front at the time t, $v_{n}\left(t\right)$ is the velocity of the vehicle behind, $\tau_{k}$ is the driver’s reaction velocity, b is the maximum deceleration of the vehicle, and $g_{n}\left(t\right)$ is the gap with the vehicle in front at time t. In Equation (2), $x_{n-1}\left(t\right),and\;x_{n}\left(t\right)$ are the locations of the vehicles in front and behind, respectively, while s refers to the minimum gap between the vehicles.

Since the safe velocity may be greater than the speed limit of the road ($v_{max}$) or greater than the speed that the vehicle can reach ($v_{n}\left(t\right)+a\Delta t$), the minimum value of these three values is set as the target velocity.

(3) $$\begin{array}{c} v_{des}=\min\left[v_{n}\left(t\right)+a\Delta t,v_{safe},v_{max}\right] \end{array}$$

where a is the acceleration, and Δt is the simulation time step.

Using the target velocity determined by Equation (3), the vehicle’s velocity at the next simulation time step is determined by Equation (4).

(4) $$v_{n}\left(t+\Delta t\right)=max[0,v_{des}-\epsilon a\eta ]$$

where ϵaη is a stochastic error term, ϵ is the velocity change, and η is a random number between 0 and 1.

Through the processes of Equations (1)–(4), the simulation was performed by changing the behavior and location of the vehicle according to the velocity determined by the simulation time step. Assuming that a vehicle driving in the simulation environment is a typical situation, the parameters of the car-following model were set to default values, and trajectory data for training on 5018 vehicles passing through unsignalized T-intersections were obtained. Additionally, a stop-and-go scenario (vehicles stop and restart at a T-intersection) and a constant-speed scenario (vehicles travel at 30 km/h throughout) were simulated, yielding trajectory data for 5225 and 5265 vehicles, respectively. The composition of the dataset per scenario is shown in Table 2. The Basic dataset was used to design the simple trajectory-prediction model. In contrast, the Stop-and-go dataset and the Constant speed dataset were employed to evaluate and improve the simple model. In all datasets, straight, left, and right turns were generated in equal proportions.

Trajectory data were recorded from the simulation at 0.1 s intervals over each vehicle’s entire driving time, capturing each vehicle’s location, velocity, acceleration, and heading. The generated trajectory dataset contains the following features:

- Vehicle ID: ID to identify each vehicle in the dataset.
- Global time: The time at which each object’s location information was recorded. Recorded every simulation time step (0.1 s) [s].
- Departure time: The time of each vehicle’s appearance in the simulation [s].
- Arrival time: Simulation end-time for each vehicle [s].
- Position X, Position Y: The vehicle’s global coordinates (X, Y) in a simulation environment [m].
- Velocity: The vehicle’s speed [m/s].
- Acceleration: The vehicle’s acceleration [m/s^2^].
- Heading: The vehicle’s heading angle.

The following features were utilized to categorize by “Vehicle ID” and “Global time” and for training and evaluation of the model: “Position X”, “Position Y”, “Velocity”, “Acceleration”, and “Heading”.

The trajectory data generated through the simulation had the following limitations. First, the generated trajectory data did not reflect the complexity of real-world driving behavior, including diverse vehicle types, driver reactions, and unexpected external influences. Second, each vehicle in the simulation only travels along the road’s centerline. Thus, while the simulated data enable controlled experimentation, they limit the generalizability to field scenarios.

### 3.2. Preprocessing

**Figure 3:**
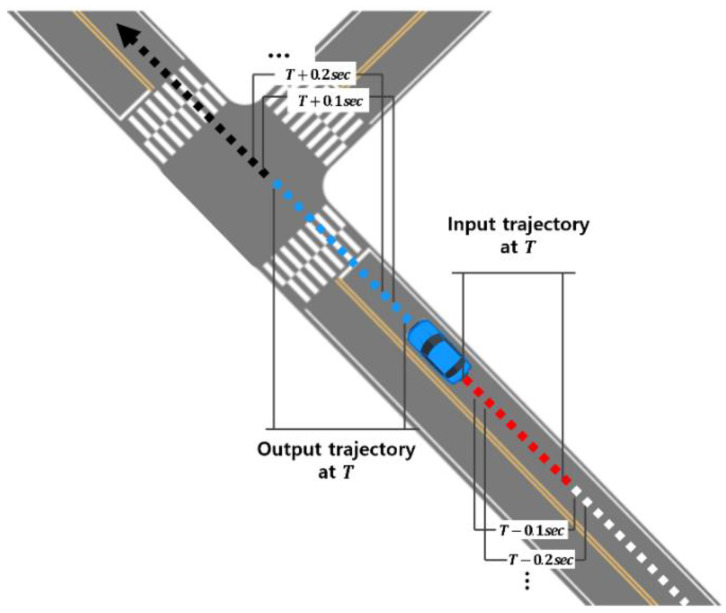
Input and output sequence for trajectory prediction.

To continuously predict a vehicle’s trajectory during its driving, it is necessary to generate input and output sequence data at each time step. Figure 3 shows how input and output trajectories are selected from the overall driving trajectory for training the trajectory-prediction model. At the current time T, when the prediction is to be performed, N past trajectory data are selected, while $\hat{N}$ corresponding future trajectory data are selected to generate one input–output trajectory sequence. Then, using a sliding window with a 0.1 s step (the simulation time step), individual sequence segments are extracted from each vehicle’s full trajectory.

**Figure 4:**
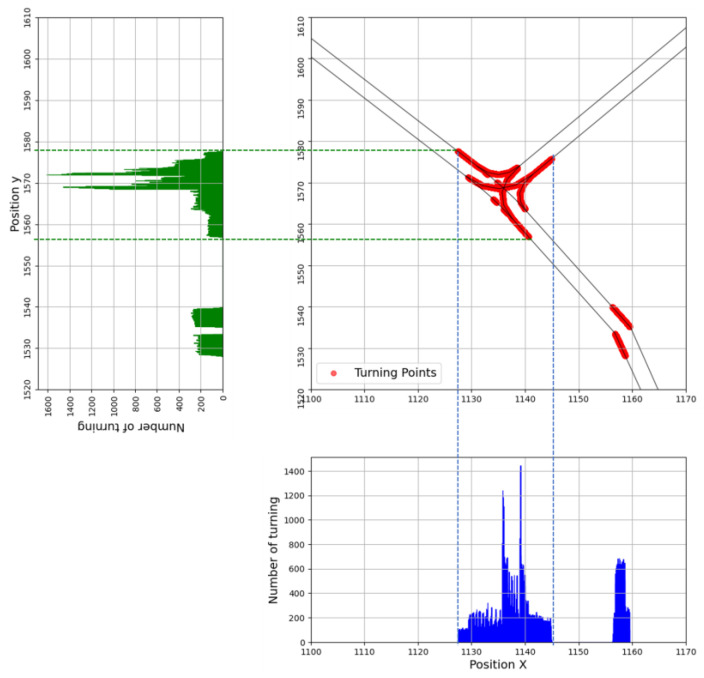
The turning segment of the trajectory.

Next, the individual input–output sequences extracted from the overall trajectory of each vehicle were separated into turning and straight driving. The heading angle (Heading) was used to categorize the sequences for each driving characteristic. At time T, the interval where the “Heading” changes by >1, compared with the “Heading” at the previous time step (T−0.1) was defined as turning, and straight driving if the value is ≤1. The locations of the turns can be found in Figure 4. In the middle graph, all the points where turns occurred were marked during the vehicle’s driving along the road’s centerline. However, in addition to turning around the T-intersection, turns around the position of (1160, 1535) in the simulation coordinates were found, which were assumed to be straight-line driving because the roadway radius of curvature caused them.

### 3.3. Transformer Model Structure

**Figure 5:**
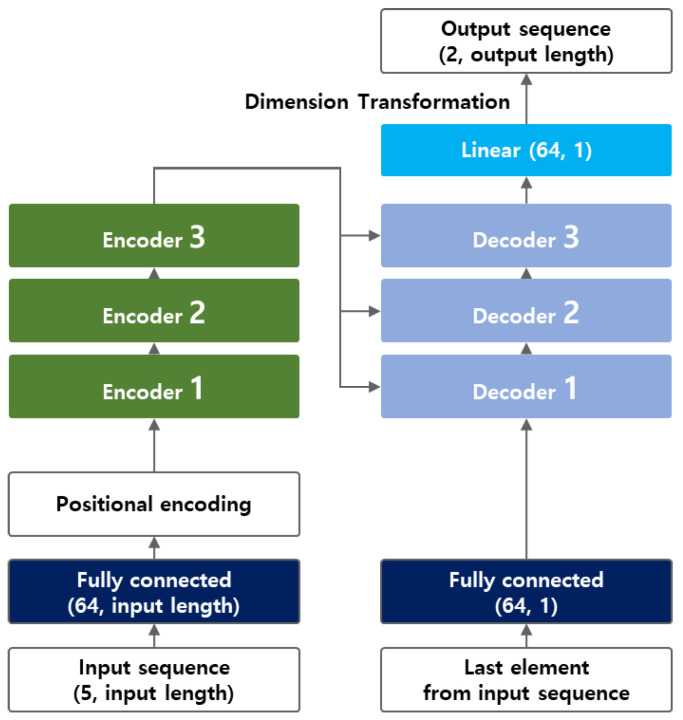
Structure of the transformer model for trajectory prediction.

Figure 5 shows the transformer model structure for this study. Before the sequence data were entered into the transformer model, a fully connected layer was constructed to serve as an embedding layer; subsequently, the data were passed through an encoding block and a decoding block, each consisting of three layers. After passing through the decoding block, the data were finally transformed into a trajectory of the desired length by passing through the fully connected layer for the output. The input data were arranged as a matrix representing 1 s (10 time-steps) with five dimensions (vehicle X and Y coordinates, velocity, acceleration, and heading). These data were first fed to the transfer layer, where they were embedded and their dimensionality was projected to 64 dimensions. This process enabled the characteristics of the input data with different units and ranges to be projected into a continuous matrix space of constant size. This also allowed the model to better understand the features inherent in the input data and allowed the self-attention mechanism to analyze the data at each point in time more accurately. Positional encoding was then applied to provide temporal context to the model. Data subjected to positional encoding were entered into an encoding block consisting of three encoders. Each encoder performed a multi-head attention calculation in which self-attention was performed in parallel. The crucial self-attention mechanism in the transformer structure was calculated as shown in Equation (5).

(5) $$Attention\left(Q,K,V\right)=softmax\left(\frac{QK^{T}}{\sqrt{d_{k}}}\right)V$$

where Q,K,and V refer to query, key, and value, respectively, while $d_{k}$ is the dimension of the key vector. The initial values of Q,K,and V are the same and are tokens of the point-in-time data currently being processed during the input sequence. Each Q,K,V weight W is updated to derive a point-in-time self-attention value.

Multi-head attentions indicate the fulfillment of multiple self-attentions in parallel at each encoder, concatenating the results from the parallel attention calculations, and then linearly projecting them back to compute the output of the encoding layer. By inputting the same Q,K,and V into multiple attention heads, the model obtains outputs from different perspectives on the same data, enriching its explanatory power. The calculation of multi-head attentions is shown in Equation (6). Four multi-head attentions were set for the trajectory prediction model.

(6) $$Multihead\left(Q,K,V\right)=Concat\left({head}_{1},{head}_{2},\ldots ,{head}_{n}\right)W$$

After passing through the encoding block, the data were fed into each of the three decoders in the decoding block. In contrast to the encoder, the decoder had a masked multi-head attention mechanism, which enabled the decoder to focus only on previous positions when generating trajectory predictions. To enhance the accuracy of the first point and maintain consistency throughout the trajectory prediction, the decoder was supplied with a vector of shape (64, 1) corresponding to the input sequence’s last value [7]. The (64, 1) vector data from the decoder was passed through a linear layer and finally converted into a trajectory prediction. Dropout was applied to each encoder and decoder to improve generalization performance and prevent overfitting. The dropout ratio was set to 0.1, a commonly used value. After the above forward propagation process, back-propagation was performed to minimize the loss between the calculated and actual values. Because model performance can vary with the choice of loss function, the model’s performance was compared by employing three different loss functions in this study.

### 3.4. Loss Function

The choice of loss function can be critical to the model’s performance. Various loss functions have been employed in previous trajectory-prediction studies [11,13,14,16]. This study sought the best-performing loss function among the three most commonly used options: MSE, smooth L1, and a combined loss.

The MSE is a loss function that calculates the average of the squared error between the predicted and actual values; it is used for training to minimize errors by penalizing larger errors during the training process. The calculation of MSE is shown in Equation (7).

(7) $$L_{MSE}=\frac{1}{N}\sum_{i=1}^{N}\left(x_{i}-{\hat{x}}_{i}\right)$$

where N is the number of points consisting of the trajectory, $x_{i}$ is the x coordinate of each point of the actual trajectory, and ${\hat{x}}_{i}$ is the x coordinate of each point of the predicted trajectory.

The smooth L1 is a combination of mean absolute error (MAE) and MSE, which calculates a different loss depending on the size of the error to ensure stable learning. It can also train the model to be less sensitive to data outliers [41]. Smooth L1 can be calculated using Equation (8).

(8) $$L_{SmoothL1}=\left\{\begin{array}{c} \frac{1}{N}\sum_{i=1}^{N}\frac{0.5{\left(x_{i}-{\hat{x}}_{i}\right)}^{2}}{\delta },\;\;\;\;\;\;\;\;\;\;\;\;\;\;\;\;\;\left|x_{i}-{\hat{x}}_{i}\right|<\delta \\ \frac{1}{N}\sum_{i=1}^{N}(\left|x_{i}-{\hat{x}}_{i}\right|-0.5\delta ),\;\;\;\;\;\;\;\;\;\;\;\;otherwise \end{array}\right.$$

where δ is the threshold to judge the magnitude of the error [41].

The y-coordinate is calculated in the same manner, and the two error values are combined and used as the overall loss value.

The combined loss was defined as the sum of the average displacement error (ADE), the final displacement error (FDE), and the root mean square error (RMSE). This loss function allows the model to be trained to minimize error on the chosen evaluation metrics.

(9) $$L_{Combined}=RMSE+ADE+FDE$$

### 3.5. Performance Evaluation Methods

Two evaluation metrics were utilized [11,14,16]. First, the ADE is a metric that evaluates the average error occurring at all points of the predicted and actual trajectories. ADE can be calculated through Equation (10):

(10) $$ADE=\frac{1}{N}\sum_{i=1}^{N}\sqrt{{\left(x_{i}-{\hat{x}}_{i}\right)}^{2}+{\left(y_{i}-{\hat{y}}_{i}\right)}^{2}}$$

where N is the number of each point consisting of the trajectory, $\left(x_{i},y_{i}\right)$ is the coordinate of each point of the actual trajectory, and $\left({\hat{x}}_{i},{\hat{y}}_{i}\right)$ is the coordinate of each point of the predicted trajectory.

Second, the FDE is a value that measures the error between the last point of the predicted trajectory and the last point of the actual trajectory and is a method of evaluating the performance of a model based only on the error regarding the last point of the trajectory. The calculation of the FDE is shown in Equation (11).

(11) $$FDE=\sqrt{{\left(x_{N}-{\hat{x}}_{N}\right)}^{2}+{\left(y_{N}-{\hat{y}}_{N}\right)}^{2}}$$

where $\left(x_{N},y_{N}\right)$ and $\left({\hat{x}}_{N},{\hat{y}}_{N}\right)$ are the coordinates of the last point of the actual trajectory and those of the last point of the predicted trajectory, respectively.

In the above evaluations, three trajectory categories were considered: an overall trajectory, a trajectory that turns at a T-intersection, and a straight trajectory. Average model performance was assessed by employing the entire predicted trajectory, whereas the turning and the straight trajectories were used to analyze the detailed performance of the model by dividing the part where the vehicle turns and where the vehicle drives straight, respectively. This distinction thoroughly evaluates how the model performs under different driving situations.

### 3.6. Training Environments and Methods

The training environment and methods for the trajectory-prediction model were as follows. The hardware specifications are Intel (USA) i9-14900KF, GeForce (USA) RTX 4080 Super, and 128 GB RAM (Samsung, Republic of Korea). The aforementioned transformer model design in the previous chapter all used the deep learning framework Pytorch [42], and the environment was PyTorch ver. 2.5.1, CUDA ver. 12.4, and cuDNN ver. 9.1.0. The setting details for training were as follows. Trajectory data were input to the model using the MinMax scaler to normalize the data to the range [0, 1]. The constant-length input and output sequence data per vehicle were generated from the original trajectory data and trained on it. In the experiment to determine the appropriate loss function, the input sequence length was fixed at 1 s and the output sequence length at 3 s, corresponding to 10 trajectory points per second. In the experiment to determine the lengths of the input–output sequences, the lengths of the input and output sequences were increased in 0.1 s increments. All trainings adopted a batch size of 256, a learning rate of 0.001 [14], and 50 epochs. The SUMO simulation’s dataset was divided into a ratio of 8:2 and utilized for training and evaluation.

## 4. Trajectory-Prediction Model Design

### 4.1. Loss Function Selection

**Figure 6:**
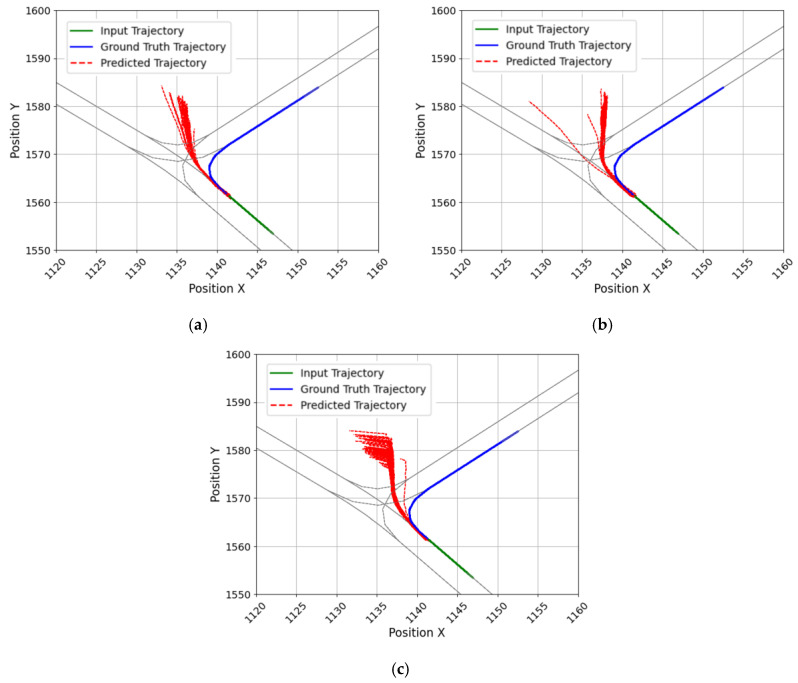
Predicted error trajectories at the intersection by loss functions: (**a**) MSE, (**b**) Smooth L1, and (**c**) combined loss.

The evaluation results of the trained models via each loss function are given in Table 3. During training, the input and output sequence lengths were fixed to 1 and 3 s, respectively. Each model was evaluated for the overall trajectory, turning, and straight driving segments as described earlier. With the exception of FDE in the overall trajectory and straight-driving case, the smallest error was achieved with the smooth L1, followed by the combined loss, and then the MSE. In the turning section, the smooth L1 was found to have the best performance among all metrics; compared with the MSE, there was a 9.7% reduction in prediction error for the ADE and an 8.2% reduction for the FDE. However, the combined loss, which combines the three metrics, yielded the lowest FDE error in both the overall and straight-driving sections. Compared to MSE, which had the most significant prediction error, the combined loss reduced the FDE by 11.2% for the overall scenario and by 17.1% for the straight-driving scenario. When training a model with the combined loss, the FDE was the lowest, which is expected to be attributable to the structure of the loss function. Because the combined loss directly includes FDE as a component, it presumably improved the FDE for straight-driving segments. However, for turning, the combined loss led to confusion, as seen in Figure 6. When using the combined loss, the endpoint of the predicted trajectory deviated in the direction of predicting straight driving, which increases the error. This is presumed to be due to the confusion between straight driving and turning during the learning process, and the optimization of the FDE with a straight path relatively close to the predicted trajectory. Based on Table 3 and Figure 6, it was judged that the smooth L1 is an appropriate loss function for training because it has high prediction performance for turning and is not overfitted for straight driving. Also, since the structure of the model was the same and only the loss function was changed, the inference time was maintained at about 1.4 ms.

### 4.2. Evaluation Performance by Input and Output Lengths

As described in this section, the models were trained, and their performance was compared while varying the sequence lengths to determine the appropriate input and output sequence lengths. A long-term input sequence increases the complexity of the model and can function as noise in the inference, consequently reducing performance [34,35]. Furthermore, the overall prediction error tends to increase as the length of the predicted trajectory increases [12,16,18]. Road infrastructure, which utilizes limited computing resources, requires the utilization of data of appropriate length to maintain a high inference rate with fewer computing resources. Accordingly, experiments were conducted to identify input–output sequence lengths that would allow high performance to be preserved. First, the models were trained with input sequence lengths ranging from 0.1 to 5 s, and their prediction performance was compared. Next, the models were trained and evaluated while varying the length of the output trajectory sequence from 1 to 10 s.

**Figure 7:**
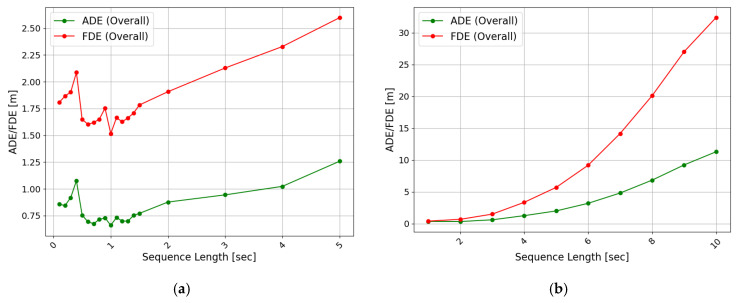
Evaluation results by input and output sequence lengths: (**a**) input sequence lengths and (**b**) output sequence lengths.

The evaluation results were as follows. First, the evaluation results of the models trained according to the length of each input sequence are shown in Figure 7a. The evaluation of each model was compared across the overall, turning, and straight sections described earlier. As the input sequence length increased from 0.1 s, the values of the ADE/FDE decreased from 0.86 and 1.85 m, respectively, and then increased after reaching 0.62 and 1.51 m at 1 s. The prediction performance remained below 0.75 and 1.75 m at input sequence lengths between 0.6 and 1.4 s. These results indicate that long-term past trajectory information does not guarantee higher accuracy in predicting future trajectories, and that there is an optimal amount of information for optimal prediction. Short-term input sequences of 0.6 s or shorter are assumed to be insufficient for the model to predict future trajectories accurately. Conversely, long-term trajectory data of 1.5 s or more are judged as noise in the prediction process and impede performance.

**Figure 8:**
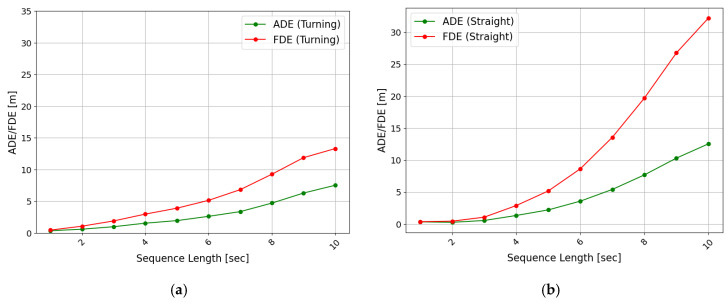
Evaluation results for turning and straight trajectory by predicted sequence lengths: (**a**) turning trajectory and (**b**) straight trajectory.

**Figure 9:**
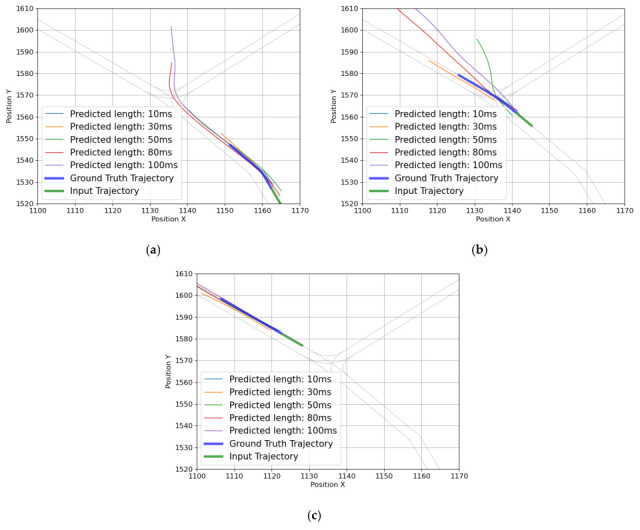
Predicted trajectory of a vehicle driving straight ahead in line with an output sequence length: (**a**) right after the appearance, (**b**) at the intersection, and (**c**) after passing the intersection.

Second, the evaluation results of the model, depending on the temporal length of the trajectory that the model attempts to predict, can be found in Figure 7b. For short prediction lengths of less than 3 s, ADE and FDE showed relatively accurate prediction performance of less than 0.6 m and 1.5 m, respectively. However, models with prediction lengths longer than 4 s started showing a rapid increase in errors. Regarding the 10 s prediction, the ADE was 11.31 m and the FDE was 32.41 m, indicating that the model’s accuracy deteriorated significantly for long-term predictions and tended to be unable to make practical predictions. Figure 8 presents the evaluation results by driving type for each predicted trajectory length. The models with output trajectory lengths of 4 s or shorter showed better prediction performance for straight driving, compared with turning; however, from 5 s onward, the prediction error for straight driving increased rapidly. For example, the evaluation results for the turning trajectory between the 4 and 5 s prediction models increased by 0.4 m (ADE) and 0.93 m (FDE), respectively. In comparison, the evaluation results for the straight trajectory increased relatively drastically by 0.87 m (ADE) and 2.28 m (FDE). The reason for this phenomenon can be presumed from Figure 9. Figure 9 shows the visualizations of the trajectories generated by the models with different predicted trajectory lengths and the actual and input trajectories. As a result of comparing the input sequence, actual trajectory, and predicted trajectory for a single vehicle randomly selected from the vehicles driving straight without turning at a T-intersection, it was found that the predicted trajectory closely matched the actual trajectory when the prediction time was short, but as the target prediction time increased, the trajectory gradually deviated from the actual driving path. Particularly, as confirmed in Figure 9a, the models performing long-term prediction in the section entering the T-intersection continuously generated a predicted trajectory that veered to the right until the turning section was reached, increasing the FDE value. Additionally, as for the long-term predicted trajectory in Figure 9a, there was a case in which the driving trajectory deviated from the road. Such a problem may be due to the model’s inability to learn information about the geometry of the road and the kinematic characteristics of the vehicle in advance [29]. Figure 9b depicts a vehicle trying to drive straight near the T-intersection; however, all the models were confused. In Figure 9c, after passing through the T-intersection, all models generated trajectories identical to the heading.

Based on the results of the input and output sequence lengths, the current trajectory-prediction model structure suggests that it is appropriate to predict trajectories using input sequences of around 1 s or less. It is presumed that predicting short-term trajectories of 3 s or less would be practically applicable.

## 5. Model Evaluation and Improvement

### 5.1. Impacts of Deceleration and Acceleration

**Figure 10:**
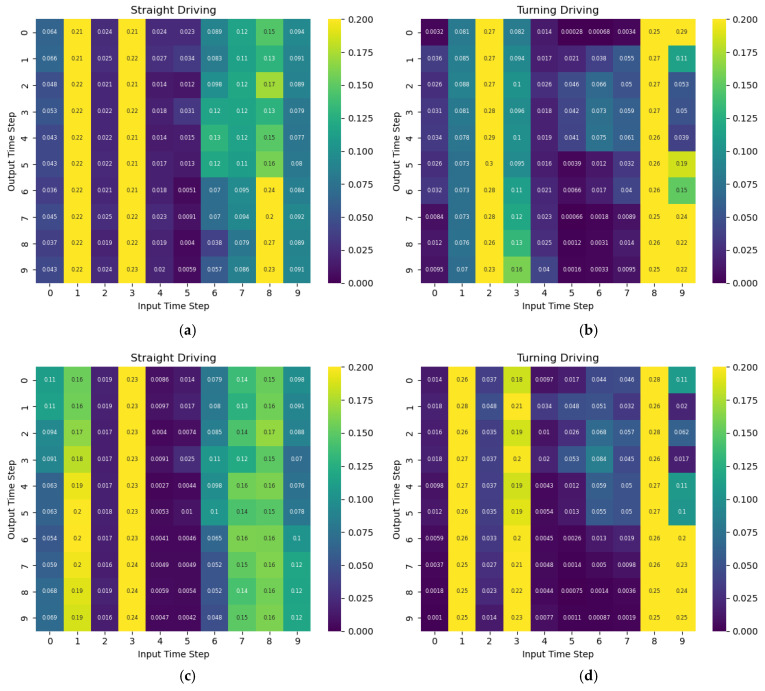

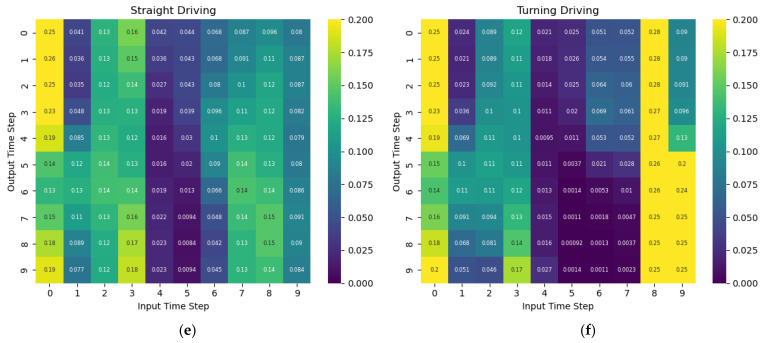
Attention weights heatmap of the model trained with the basic data for each driving behavior: (**a**) straight of basic dataset, (**b**) turning of basic dataset, (**c**) straight of stop-and-go dataset, (**d**) turning of stop-and-go dataset, (**e**) straight of constant speed dataset, and (**f**) turning of constant speed dataset.

In this section, new types of trajectory data, which had been absent or rare in the existing training set, were constructed and evaluated to verify the generalization performance of the transformer model. Two types of trajectories were used in the evaluation. The first type is a stop-and-go scenario, which departs from the normal driving pattern and repeatedly involves intentional stops and restarts near the intersection. The second type is a uniform motion scenario in which the vehicle moves constantly from entry to exit. Since both scenarios have different characteristics from the distribution of the existing training data, they are appropriate for confirming the generalization performance of the model. Table 4 shows the evaluation results of the model that was additionally trained on each scenario dataset in Table 1. Figure 10 presents a heatmap of attention weights for the straight driving and turning sections using the model trained on the basic dataset. Figure 10a,b shows that the model focuses on the initial time-step inputs when predicting straight driving, but shifts attention to later inputs when predicting turning driving. Stop-and-go data also showed similar attention distributions, but the attention to time step 8 decreased when predicting straight driving. In the constant speed data, a decrease in attention to the initial inputs was observed in both straight and turning driving.

**Figure 11:**
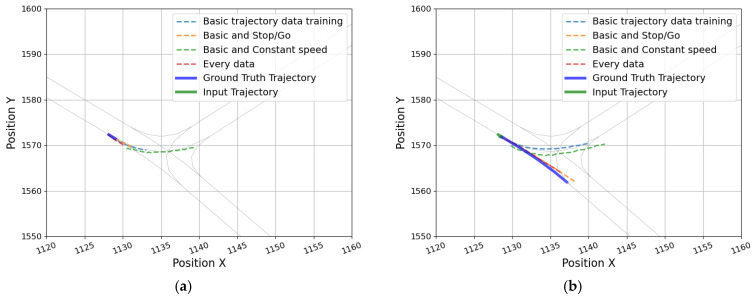
Predicted trajectories of each model for a stop-and-go vehicle: (**a**) when the vehicle stopped and (**b**) after restart.

As Table 4 shows, for the model trained only on the basic dataset, the overall prediction error on the stop-and-go dataset test increased dramatically, compared to its performance on the basic data evaluation. Specifically, in the straight driving portion of the stop-and-go test, ADE and FDE increased to 1.61 m and 3.19 m, respectively. In contrast, the error was smaller for the turning maneuver of the stop-and-go vehicle compared to the basic data evaluation. This is because, as shown in Figure 11, when a vehicle pauses at a T-intersection to make a turn, the predicted trajectory becomes very short temporarily. However, as confirmed in Figure 11, when the vehicle pauses and restarts to drive straight, the error seems larger because the predicted trajectory remains in the left-turn direction. This implies that the transformer model can predict some dynamic trajectories of decelerating, stopping, and re-accelerating without additional training; however, the confusion between straight and turning at T-intersections is maintained.

**Figure 12:**
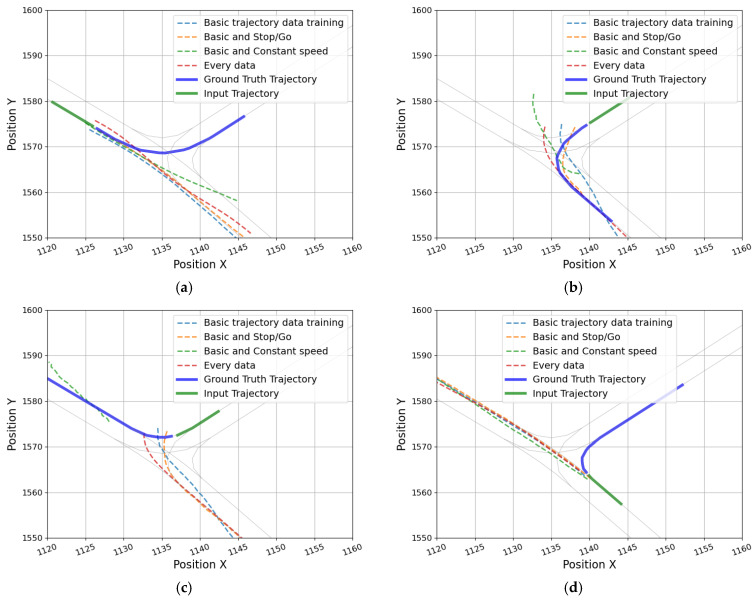
Incorrectly predicted trajectories of each model for the constant-speed driving vehicles near the intersection: (**a**,**b**) left turn and (**c**,**d**) right turn.

The trajectory prediction models were additionally trained and evaluated with scenario-specific data. First, the model, which was additionally trained with the stop-and-go data, improved the prediction error for stop-and-go data. Especially, the reduction in error was considerable for the straight-driving section (ADE: 1.61 → 0.75 m, and FDE: 3.19 → 1.66 m). And as shown in Figure 11, the predicted trajectories at the stop and the restart point were generated similarly to the actual trajectory. However, for evaluating the basic data, the ADE and FDE were increased by 0.24 m and 0.32 m, respectively; even for constant-speed data, the error per evaluation metric increased by 0.12–0.2 m. These results suggest that a model that is overfitted to certain types of driving patterns may be weaker at predicting trajectories for other driving scenarios. Second, the model, which was additionally trained with the constant speed data, showed almost no improvement in accuracy for the constant speed data. Third, the evaluation results of the model that learned all types of driving data showed that while the overall prediction performance was expected to increase, the performance of the model was inferior to that of the model trained by reinforcing each driving characteristic data. This outcome is attributable to learning all the data under various situations, which confused the model due to the increased number of predictable trajectories when entering a T-intersection. Finally, for the turning of constant speed, all models confused the direction of the predicted trajectory, and the FDE was more than 3 m. Figure 12 presents the incorrectly predicted trajectories of each model for the four types of turning among the constant-speed data. Considering the above results, additional training for the scenario-specific data led to overfitting. Therefore, it can be presumed that structural improvement of the model is necessary instead of simply securing data diversity.

### 5.2. Impact of the Additional Feature Data

In this section, the impact of the trajectory data feature extension strategy on prediction performance was evaluated and compared with a model that learned only the five features consisting of the simulation-generated trajectory data. The trajectory data generated through simulation make it possible to calculate the variations representing the position and driving characteristics at past time, from the existing five features indicating the driving characteristics of the vehicle at each point in time. This study added four features (X change, Y change, distance, and heading change) to locations and directions by comparing the current feature data with the feature data from 0.1 s ago.

- X change, Y change: The variations in the current coordinate in the simulation environment, compared with the previous time step [m].
- Distance: The distance between the current and previous point coordinates in the simulation environment [m].
- Heading change: Variations in the current heading, compared with the previous time step heading.

The model was trained with trajectory data consisting of nine features, including four newly computable features, and compared to a model that learned only five features. Also, a model was trained with only seven features, excluding the X and Y coordinates, and the evaluation results were compared. The trajectory-prediction model examined in the previous section preferentially generated turning trajectories near T-intersections, which is presumed to be highly correlated with the vehicle’s position coordinates. Thus, a dataset with only seven features, excluding the X, Y coordinates, was constructed, a model was trained on this dataset, and its performance was compared to the other models.

Table 5 shows a comparison of the prediction performance of the same structured transformer model with three different input feature configurations. The model trained with all nine features showed lower performance than the model trained with five basic features overall. The ADE for the overall trajectory increased by 0.24 m, while the FDE increased by 0.32 m. The evaluation errors for turning and straight driving also increased, particularly with the largest increase in FDE for turning (0.76 m). This indicates that introducing variation features can induce redundant information or learning confusion into the model and does not necessarily lead to improved performance. In particular, because the transformer’s self-attention already captures temporal patterns on its own, explicitly feeding it additional variation features may be redundant. The model trained on the remaining seven features, excluding the X, Y coordinates from the nine features, had the worst performance of the three models (ADE 5.36 m, FDE 5.63 m). The error was found to be consistently high in all sections, especially with an FDE of 5.69 m in the straight-driving section, which is five times higher than that of the other models. This demonstrates that variation features alone cannot compensate for the lack of structural information such as absolute position, direction, and velocity. In other words, variation can act as supplementary information but cannot create predictive power on its own without basic trajectory position information. And the inference time of the models for each feature had a maximum difference of 0.169 ms. Although the inference time increased as the number of features increased, it is shorter than the 100 ms interval at which the actual trajectory data are generated, so it is judged that there will be no time delay problem when used in an edge computing environment.

These results indicate that the addition of variation features over time does not always improve performance in trajectory-prediction models and may even exhibit degradation in performance depending on the model structure and learning method. The usefulness of variation features may be limited in model structures that internally process temporal information, such as transformers. This result implies that careful choices based on domain knowledge are needed when constructing input features, and that simple feature extension does not guarantee predictive power.

## 6. Conclusions

In this study, the basic structure of a transformer model was designed that can predict vehicles’ trajectory by using past trajectory information of vehicles entering and exiting the turning section of T-intersections, and experiments were conducted to explore the direction in which it can be improved and utilized efficiently. The following conclusions were drawn. First, smooth L1 loss is the most appropriate loss function for training the trajectory-prediction model, especially for reducing the prediction error in the turning section. Second, it was found that a large amount of past trajectory information does not guarantee higher accuracy of future trajectory prediction, and that there is an optimal amount of information for optimal prediction. Since a 1 s sequence length seems to provide an optimal balance between contextual richness and noise minimization, the usage of such a sequence length is advantageous to achieve the best model performance while maintaining computational efficiency. Third, evaluations based on vehicle-driving scenarios suggest that generalized performance can be expected without a large amount of training under specialized situations. However, it was confirmed that driving patterns that are considered simple, such as driving at a constant speed, can be confusing for the transformer model. To improve this problem, a structural improvement may be needed to create and integrate prediction agents specialized for straight driving and turning separately. Fourth, it was confirmed that the extension of feature data does not always improve prediction performance. Since the addition of features related to temporal correlation in trajectory data degrades performance, structural improvements to the model may be more effective than data preprocessing. Finally, the proposed models showed high computational efficiency with an inference speed of less than 1.6 ms in a GPU environment. This is considered suitable for real-time edge computing because the inference time is shorter than the time it takes for data to be generated for each trajectory point.

This study proposed a transformer-based trajectory prediction model for vehicles at urban T-intersections and adjacent roads, which can help manage traffic flow and prevent accidents. Although the proposed model improved average prediction accuracy, it was observed that in certain cases the predicted trajectory deviated from the ground truth. This limitation suggests that for safety-critical applications such as collision avoidance, even higher accuracy and reliability are required. Therefore, future research will be conducted to improve the model’s performance in difficult-to-predict scenarios to expand its practical applicability. Additionally, given the clear limitations of the model’s structure and training data, further research is needed. In terms of data for model training, it may be necessary to utilize road geometry information and data on the position relationships among individual vehicles. Furthermore, to overcome the generalization limitations of simulation-only training, future research will incorporate real-world trajectory data collected from sensor-embedded infrastructure at urban intersections. These data will include interactions between multiple vehicles (e.g., accounting for collision avoidance and right-of-way rules) and varying environmental conditions. This will support domain-adaptive training and further validate the model’s practical applicability. In terms of structural improvements to the model, it may be necessary to develop a model that can infer the driving intention of the vehicle in advance and predict the trajectory, or a model that can separately train and ensemble a specialized model for turning and straight driving. Finally, the deployment of this trajectory prediction system should consider ethical implications, including the privacy of collected vehicle data and the safety of its use. Ensuring that the system augments driver decision-making without infringing on privacy or safety is crucial for public acceptance. The trajectory-prediction models developed through such studies are expected to contribute to the safe route planning of vehicles in complex urban road environments and improve the efficiency of traffic management systems.

## Figures and Tables

**Table 1:** Comparison of three major architectures for trajectory prediction.

Model Type	Advantages	Disadvantages	
RNN	- Simple structure - Sequential data training	- Gradient vanishing - Difficult to parallelize	[3,4,5,6]
LSTM/ GRU	- Improved long-term dependencies	- Increased computational cost than RNNs - Difficult to parallelize	[7,9,28]
Transformer	- Parallel sequence processing - Effectively captures long-term dependencies - Flexible to variable input length	- Complex structure	[11,12,13,14,15,16,17,18]

**Table 2:** The trajectory datasets by driving pattern.

Trajectory Dataset	Driving Speed			Number of Vehicles
	Basic	Stop-and-Go	Constant	
Basic	4571	447	0	5018
Stop-and-go	0	5225	0	5225
Constant speed	0	0	5265	5265

**Table 3:** Evaluation results of models for each loss function [m].

Metric	MSE		Smooth L1		Combined	
	ADE	FDE	ADE	FDE	ADE	FDE
MSE	0.667	1.671	1.084	2.059	0.63	1.24
Smooth L1	0.615	1.506	0.979	1.891	0.576	1.095
Combined	0.635	1.483	0.995	2.017	0.597	1.027
Inference time [ms]	1.428		1.435		1.456	

**Table 4:** Evaluation results by driving characteristics [m].

Train Data	Test Data	Overall		Turning		Straight	
		ADE	FDE	ADE	FDE	ADE	FDE
Basic	Basic	0.62	1.51	0.98	1.89	0.58	1.09
	Stop-and-go	1.33	2.73	0.64	1.63	1.61	3.19
	Constant speed	0.98	2.44	1.40	3.55	0.72	1.75
Basic + Stop-and-go	Basic	0.86	1.83	1.05	2.55	0.71	1.26
	Stop-and-go	0.78	1.72	0.83	1.85	0.75	1.66
	Constant speed	1.13	2.59	1.52	3.75	0.88	1.87
Basic + Constant speed	Basic	0.76	1.79	1.19	2.14	0.72	1.37
	Stop-and-go	1.57	2.92	0.93	1.99	1.84	3.31
	Constant speed	1.01	2.37	1.33	3.36	0.80	1.75
Basic + Stop-and-go + Constant speed	Basic	0.86	1.92	1.19	2.19	0.76	1.16
	Stop-and-go	0.91	2.14	0.87	2.19	0.93	2.13
	Constant speed	1.16	2.59	1.46	3.62	0.97	1.94

**Table 5:** Evaluation results from additional feature training [m].

Train/Test Feature	Overall		Turning		Straight		Inference Time [ms]
	ADE	FDE	ADE	FDE	ADE	FDE	
Position X, Position Y, speed, acceleration, heading	0.62	1.51	0.98	1.89	0.58	1.10	1.435
speed, acceleration, heading, X change, Y change, distance, heading change	5.36	5.63	4.97	5.56	5.67	5.69	1.452
Position X, Position Y, speed, acceleration, heading, X change, Y change, distance, heading change	0.86	1.83	1.05	2.65	0.71	1.20	1.604

## Data Availability

The data presented in this study are available on request from the corresponding author.
